# Intergenerational trauma in Poland

**DOI:** 10.1016/j.lanepe.2025.101379

**Published:** 2025-07-01

**Authors:** The Lancet Regional Health – Europe

The number of active armed conflicts globally has increased sharply in recent years, reaching levels not seen since World War 2, with the World Economic Forum ranking state-based armed conflict as the highest global risk in 2025, surpassing extreme weather events and misinformation. In 2024 alone, 1 in 8 people were estimated to have been exposed to conflict, and an estimated 2 billion people were exposed to the trauma of war, a figure that is likely to grow given the ongoing wars in Ukraine, Gaza, and now between Israel and Iran. While the physical toll of war is immediate and visible, its deeper scars are often psychological—and, crucially, intergenerational.

Poland, with its complex wartime history, highlights the enduring reality of intergenerational trauma, where the psychological and physiological effects of past atrocities are passed down through generations.

After losing approximately 17% of its population during World War 2—the highest proportional loss of any country involved—Poland continues to carry the burden of trauma. It is, therefore, not surprising that Poland reports one of the highest rates of post-traumatic stress disorder (PTSD) in the world. Children and grandchildren of Polish Holocaust survivors are at higher risk of PTSD, depression, and anxiety, as well as having increased prevalence of health problems such as cancer, heart disease, and chronic pain. The manifestations of trauma in successive generations vary but often include feelings of shame, suicidality, substance abuse, and heightened reactivity to stress.

Poland faces persistent challenges in addressing this invisible inheritance. Mental health care remains under-resourced, with only nine psychiatrists per 100,000 people, and stigma continues to deter many from seeking help—60% of those who report needing support do not receive it. Research has shown that after extreme trauma, people can develop one of three adaptation styles: victim (eg, emotional instability and fear); numb (eg, maintaining conspiracy of silence and low tolerance to stimuli); and fighter (eg, drive to achieve and prohibiting weakness). When Polish families were interviewed about their World War 2 stories, themes of strength, bravery, and patriotism frequently emerged in narratives describing Polish identity. However, silencing either the entire story or key details was the most common pattern. Such behaviour can lead to trauma denial or a lack of social recognition and validation, reducing emotional openness and cohesion within the family, and ultimately perpetuating intergenerational trauma.

A culture of silence around trauma can stem from living under repressive regimes, where fear-based survival messages—such as “don't ask for help, it's dangerous”—are internalised and passed down through generations. In Poland, for example, the transition from German to Soviet occupation forced members of the Home Army, a World War 2 resistance group, to suppress their trauma for fear of persecution or execution. While such messages might serve as survival strategies during conflict, they can become barriers to healing in peacetime, preventing trauma survivors from seeking support.

Encouragingly, Poland has initiated systemic efforts to address these longstanding issues. The integration of mental health services into primary care through the creation of Mental Health Centres—supported by WHO's Mental Health Gap Action Programme—has improved continuity of care, reduced unnecessary psychiatric hospitalisations, and enhanced support for vulnerable populations, including Ukrainian refugees. This model sets a precedent for regional leadership in trauma-informed, community-based care.

Scientific advances in epigenetics have begun to clarify how trauma may be biologically inherited. Reduced methylation of the FKBP5 gene—first observed in Holocaust survivors and later in Syrian refugees—has been linked to increased stress sensitivity and dysregulation of the hypothalamic–pituitary–adrenal axis, the body's key stress-response system. More recently, an epigenome-wide association study found DNA methylations linked to war-related violence in three generations of Syrian refugees.

The lessons from Poland are clear: intergenerational trauma is not a distant concern. It is a public health issue rooted in real biological and social mechanisms, and it is poised to grow amid current and future conflicts.

To address the needs of populations affected by intergenerational trauma, governments must prioritise long-term mental health infrastructure and trauma-informed policymaking. Without meaningful investment—across research, policy, education, and health systems—trauma will continue to cascade through generations. The scientific and clinical communities need to intensify research into the transmission, prevention, and treatment of intergenerational trauma.

The cost of inaction extends beyond present suffering, perpetuating a legacy of trauma and pain that lasts long after the war has ended.

